# Adverse reactions to mRNA coronavirus disease 2019 (COVID-19) vaccine for severe acute respiratory syndrome coronavirus 2 (SARS-CoV-2) in 576 medical staff

**DOI:** 10.20407/fmj.2021-009

**Published:** 2021-11-25

**Authors:** Masamichi Hayashi, Sayako Morikawa, Yusuke Goto, Takazumi Yoshida, Yutaro Kimura, Kazuyoshi Imaizumi

**Affiliations:** 1 Department of Internal Medicine, Division of Respiratory Medicine, Fujita Health University, School of Medicine, Okazaki, Aichi, Japan; 2 Department of Respiratory Medicine I, Fujita Health University, School of Medicine, Toyoake, Aichi, Japan; 3 Department of Respiratory Medicine II, Fujita Health University, School of Medicine, Nagoya, Aichi, Japan

**Keywords:** SARS-CoV-2, COVID-19, mRNA vaccine, Adverse reaction, Anaphylaxis

## Abstract

**Objectives:**

Various measures have been taken to curb the COVID-19 epidemic, but their effect has been limited. Vaccines are expected to make a definite improvement. In Japan, vaccination began in February 2021. However, there are very few reports of adverse reactions to mRNA vaccines for the new coronavirus (SARS-CoV-2) in Japanese people. Therefore, adverse reactions in 576 vaccinated medical staff at the author’s hospital were investigated.

**Methods:**

The subjects were 576 medical staff who wished to receive the new coronavirus (SARS-CoV-2) vaccine. The first vaccination was performed with Pfizer’s new coronavirus mRNA vaccine (commercial name: Comirnaty intramuscular injection) from March 8 to March 15, 2021. The second vaccination was performed from March 29 to April 5, 2021, at an interval of more than 21 days from the first vaccination.

**Results:**

Adverse reactions were seen in six subjects in the first vaccination. There was dyspnea in one subject, arthralgia in one subject, fever/malaise in three subjects, and left axillary pain and lymphadenopathy in one subject. One subject had a reaction at the vaccination site that needed to be addressed. At the second vaccination, adverse reactions were observed in 64 subjects. There was fever in 58 subjects, malaise in 21 subjects, arthralgia in 12 subjects, aching pain in 11 subjects, headache in six subjects, chills in six subjects, nausea in three subjects, redness in two subjects, dizziness in two subjects, hives in two subjects, swelling in two subjects, cough in one subject, and itching in one subject (some had multiple adverse reactions). Fever was observed in the range of body temperature from 37.4 to 38.9 degrees.

**Conclusions:**

Most of the adverse reactions to the COVID-19 vaccine were mild, and no serious anaphylaxis was observed. Vaccination was considered perfectly feasible if attention is paid to adverse reactions.

## Introduction

Coronavirus disease 2019 (COVID-19) emerged in Wuhan, China, and very rapidly spread all over the world. There have been several peaks in the epidemic, but there is no sign that it will end even now.^1^

In Japan too, it spread in a short period of time and occurred outbreak on the Diamond Princess, February 3, 2020, and Japan is now experiencing the fourth wave.^2,3^

Various measures have been taken to control the epidemic, but the effects have been limited. Vaccines are expected to make a definite improvement.

Vaccination is expected to reduce disease severity, reduce the mortality rate, and end the infection by conferring herd immunity.

In the rest of the world, vaccination had already started in December 2020. In Japan, Pfizer-BioNTech’s new coronavirus vaccine (commercial name: Comirnaty intramuscular injection) was approved as a special case based on the result of a Phase 3 study in the United States,^4^ and priority vaccination of 40,000 health care workers finally took place starting in February.

It is said that 60–70% of the population needs to be vaccinated at least once to obtain herd immunity. Vaccination must be done urgently, but as of April 10, 2021, Japan was only able to vaccinate 0.87% of the population, unfortunately lagging far behind leading countries such as the United States, China, India and the United Kingdom.

There have been many reports of adverse reactions from the United States, but few reports from Japan. Various countries also have reported that there are more adverse reactions at the second vaccination, and there have been similar reports in Japan for health care workers who were vaccinated first.

Therefore, on this occasion, as Pfizer’s new coronavirus vaccine started to be given on March 8, 2021, to 576 medical staff who wished to receive a vaccination at the author’s hospital, the authors decided to investigate adverse reactions.

## Methods

The subjects were 576 medical staff who wished to receive the new coronavirus vaccine.

The first vaccination was performed with Pfizer-BioNTech’s new coronavirus vaccine (commercial name: Comirnaty intramuscular injection) from March 8 to March 15, 2021.

One vial containing the vaccine was divided into five doses (for five subjects), and a 1 ml syringe with a 27 G needle was used. An alcohol cotton swab was used for disinfection of the skin, and those with hypersensitivity to alcohol were disinfected with chlorhexidine. A 0.3 ml intramuscular injection was then given to the triceps brachii muscle of the non-dominant shoulder.

The second vaccination was performed from March 29 to April 5, 2021, at an interval of more than 21 days from the first vaccination.

Subjects who are unsuitable for vaccination are specified in the Vaccination Regulations and the New Coronavirus Provisional Vaccination Guidelines, as follows:

1)  Those who have received other vaccinations related to coronavirus disease
2)  Those who have an obvious fever
3)  Those who have a known history of anaphylaxis due to the components of the vaccination solution
4)  In addition to those who fall under the above, those who are unsuitable for vaccination for other reasons.

To deal with possible anaphylaxis, according to the guidelines of the US Centers for Disease Control and Prevention (CDC), those who had a severe allergic reaction, and those who had an immediate allergic reaction to a vaccine or injection, were treated as persons requiring special care with regard to vaccination, and were followed-up at the vaccination site for 30 minutes after vaccination, whereas other persons were followed up for 15 minutes.

To ensure the system could respond in the event of anaphylaxis, emergency carts equipped with items necessary for emergency treatment such as adrenaline preparation, as well as stretchers and beds for transportation were prepared.

The vaccine was prepared by the pharmacist. Two doctors checked the vaccination medical interview sheets, two nurses vaccinated the subjects, and one nurse followed them up. Four clerks checked and received the interview sheets. At the vaccination site, chairs for 30 subjects were distanced from each other, and follow-up was performed while the subjects were sitting on the chairs.

Adverse reactions after follow-up were reported to the superiors of each department by a self-reporting system, and the Infection Control Team (ICT) asked detailed questions about symptoms. Cases that were considered to be adverse reactions were reported to the Ministry of Health, Labour and Welfare in a “suspect adverse reaction report.”

## Results

The average age of vaccination applicants was 34.9 years (21–69 years, median 32 years), comprising 396 women and 180 men (Table 1). Their previous medical histories were hypertension (15), hyperlipidemia (7), endometriosis (4), diabetes (4), dysmenorrhea (3), hyperthyroidism (3), ovarian cyst (2), depression (2), glaucoma (2), epilepsy (1), systemic lupus erythematosus (SLE) (1), prostatic hypertrophy (1), rheumatoid arthritis (1), dystonia (1), obstructive sleep apnea syndrome (OSA) (1), migraine (1), Crohn’s disease (1), gastroesophageal reflux disease (1), and cholelithiasis (1) (Table 1). A total of 36 subjects had a history of allergies, including those with hay fever (7), bronchial asthma (6), atopic dermatitis (5), and allergic rhinitis (2), or allergy to shellfish (3), or eggs and dairy products (2). Others had an allergy to yellowtail, peach, kiwi, shiitake mushroom, or buckwheat (one subject each), or to oral medications such as antibiotics (five subjects). There was no history of serious allergic symptoms such as anaphylaxis, but some subjects had an adverse reaction of fever to the influenza vaccine (3), or an adverse reaction of fever to the cervical cancer vaccine (2) (Table 1).

At the first vaccination, adverse reactions were seen in six subjects (1.0%), specifically dyspnea (1), arthralgia (1), fever/malaise (3), and left axillary pain and lymphadenopathy (1) (Table 2).

The employee who complained of dyspnea was a 48-year-old woman with a history of bronchial asthma, and 10 minutes after vaccination reported dyspnea, malaise, and headache. Her blood pressure was 133/67 mmHg, pulse rate was 65/min, SpO_2_ was 99%, and a mild rash was observed in the corner of the mouth. Although it did not correspond to anaphylaxis in the Brighton classification,^5^ after fluid replacement and administration of antihistamines, symptoms improved rapidly.

At the second vaccination, adverse reactions were observed in 64 subjects (11.1%), specifically fever (58), malaise (21), arthralgia (12), aching pain (11), headache (6), chills (6), nausea (3), redness (2), dizziness (2), hives (2), swelling (2), cough (1), and itching (1) (some had multiple adverse reactions) (Table 3).

Only one subject required treatment at the vaccination site, who received infusion treatment for dyspnea. This employee only was not given the second vaccination for safety reasons.

Adverse reactions after the second vaccination were observed in 64 subjects (11.1%), an increase of 10.6 times from the first adverse reactions in six subjects (1.0%).

Fever was observed from the evening of the day of vaccination to the next day with a body temperature ranging from 37.4 to 38.9 degrees.

## Discussion

Adverse reactions, especially fever, increased after the second vaccination in medical staff at the author’s hospital. Currently in the corona pandemic, if there is a fever, medical staff are suspected of having an infectious disease and cannot go to work. If a large number of medical staff cannot go to work, the hospital inevitably cannot function properly. In order to prevent this, the author’s hospital used antipyretic analgesics for the medical staff in advance for expected fever and arthralgia due to the second vaccination, and thus made it possible for them to go to work if the fever resolved and symptoms improved the next day. It was thought that in the future, other medical institutions would need to take similar measures.

The US CDC used a vaccine adverse reaction reporting system called v-safe to monitor adverse reactions in 21,843,033 people every day for the first seven days after vaccination, weekly up to six weeks, and then three, six, and 12 months later.^6^ It was reported that, as of January 14, 2021, the adverse reactions were aching pain (70.7%), malaise (33.4%), headache (29.4%), myalgia (22.8%), chills (11.5%), fever (11.4%), swelling (11.0%), arthralgia (10.4%), and nausea (8.9%).^6^ It was further reported that the adverse reactions to the first vaccination with Pfizer-BioNTech vaccine were aching pain (67.7%), malaise (28.6%), headache (25.6%), myalgia (17.2%), chills (7.0%), fever (7.4%), swelling (6.8%), arthralgia (7.1%), and nausea (7.0%), and the adverse reactions to the second vaccination were aching pain (74.8%), malaise (50.0%), headache (41.9%), myalgia (41.6%), chills (26.7%), fever (25.2%), swelling (26.7%), arthralgia (21.2%), and nausea (13.9%),^6^ so adverse reactions to the second vaccination were worse.

The Vaccine Adverse Event Reporting System (VAERS) jointly operated by CDC and FDA in the United States reported adverse reactions in 7,307 people vaccinated with Pfizer-BioNTech vaccine. Overall, as of January 18, 2021, adverse reactions were headache (21.2%), malaise (16.3%), dizziness (15.2%), nausea (13.9%), chills (13.5%), fever (13.2%), aching pain (13.1%), pain at the injection site (9.8%), pain in the extremities (8.4%), and dyspnea (7.3%).

At the author’s hospital, probably because of the self-reporting system, there were few predicted adverse reactions such as aching pain and swelling at the injection site and malaise. The most frequent adverse reaction, fever, was observed as in the reports of other countries. No anaphylaxis was observed.

In the United States, from December 14 to December 23, 2020, with 1.9 million vaccinations, 21 cases of anaphylaxis were reported. This corresponds to 11.1 reports when converted to one million vaccinations.^7^

Also in the United States, between December 14, 2020, and January 18, 2021, with 9.94 million vaccinations, 47 cases of anaphylaxis were reported. This corresponds to 4.7 reports when converted to one million vaccinations. 74% occurred within 15 minutes after vaccination, 90% occurred within 30 minutes after vaccination, and 80% were people with a history of allergies.^8,9^

In the United Kingdom, from December 9, 2020, to February 28, 2021, with 11.5 million vaccinations, 214 cases of anaphylaxis were reported. This corresponds to 18.6 reports when converted to one million vaccinations.^10^

In a study of 64,900 employees of the U.S. health care system, with 25,929 vaccinations of Pfizer vaccine, allergic reactions were self-reported in 506 cases (1.95%), and anaphylaxis in seven cases (2.7 per 10,000).^11^

The frequency of anaphylaxis due to vaccinations is generally 1.3 per million, so there is concern that it is much higher than for other vaccines.^12^

In Japan, based on the Immunization Law and the Pharmaceuticals and Medical Devices Act, medical institutions that have observed adverse reactions must promptly report suspected adverse reactions to the Pharmaceuticals and Medical Devices Agency (PMDA), and share information with the Ministry of Health, Labour and Welfare.

In Japan, from February 17 to March 11, 2021, 181,184 vaccinations were given, and 37 cases of anaphylaxis were reported. This corresponds to 20.4 per 100,000, which would be 204 cases when converted to one million vaccinations.^13,14^

The number of reports in Japan appears to be very large compared to Europe and the United States, but since the number of reports at the present time is the number from medical institutions, the information must be closely examined, and the number may not correspond to anaphylaxis in the Brighton classification.

According to the guidelines of the Japanese Society of Allergology, no adjuvant or preservative is added to the COVID-19 vaccine, but the Pfizer and Moderna mRNA vaccines contain polyethylene glycol (PEG), which is used to maintain water solubility of the lipid double membrane that forms lipid nanomolecules enveloping the active ingredient mRNA, and this is thought to be the cause of anaphylaxis. However, the possibility of specific IgE production for double-stranded RNA, which is the main component of the vaccines, has not been ruled out at this time.^15^

There will probably be more reports of adverse reactions in Japan in the future, but vaccination has already begun nationwide without waiting for these reports.

mRNA vaccines have been developed and manufactured using techniques different from conventional vaccines, and there are many unclear points regarding adverse reactions.

It has been reported that anaphylaxis, which is severe hypersensitivity, occurs more frequently than with conventional vaccines, but the symptoms of anaphylaxis and its treatment are the same as for that due to other causes, and subjects who experience anaphylaxis due to the new coronavirus vaccine do recover with appropriate treatment.

Vaccinations for health care workers are scheduled to be given on a wider scale, and vaccinations for some elderly people started on April 12, 2021.

Since there are few reports of adverse reactions to new coronavirus vaccines in Japan, the reports of adverse reactions at the author’s hospital will furnish very important information for future vaccinations.

This report will hopefully be of some help to medical staff who are responding in the field so that they can take appropriate measures without disruption of the medical system.

### Study limitations

These adverse reactions are those of a single-center study and are self-reported. In order to obtain more detailed information, a monitoring system should be constructed using telephones and the Web as in the United States.

In the future, the number of cases should be increased, more reports of adverse reactions collected, and joint research undertaken with other institutions.

## Conclusion

As regards COVID-19 vaccines, many adverse reactions were experienced at the second vaccination, but most of them were mild, and serious anaphylaxis was not observed.

As reported from Europe and the United States, it was concluded that vaccination is perfectly feasible if attention is paid to adverse reactions.

From now on, the latest adverse reaction information should always be reviewed, and vaccination performed appropriately in the light of that information.

## Figures and Tables

**Table1:** Characteristics of subjects before vaccinations

N=576	
Age, mean(range, median)	58.8 (21–69, 32)
Sex	Female 396, Male 180
Past history	Hypertension (15), Hyperlipidemia (7), Endometriosis (4), Diabetes (4), Dysmenorrhea (3), Hyperthyroidism (3), Ovarian cyst (2), Depression (2), Glaucoma (2), Epilepsy (1), SLE (1), Prostatic hypertrophy (1), RA (1), Dystonia (1), OSA (1), Migraine (1), Crohn’s disease (1), GERD (1), Cholelithiasis (1)
Allergies	Allergic rhinitis (9), Bronchial asthma (6), Atopic dermatitis (5), Antibiotics (5), Shell (3), Egg·Milk (2), Urticaria (2), Fish (1), Peach (1), Kiwi (1), Mushroom (1), Buckwheat (1)
Allergic reactions or Anaphylaxis	Fever after Influenzae (3) or HPV (2) vaccines

**Table2:** Self-reported symptoms of adverse reaction to first dose of mRNA COVID-19 vaccines

N=6 (1.0%)			
	Symptoms	Grade	Outcome
Case 1	Dyspnea	mild	Resolved after taking antihistamine
Case 2	Joint pain	mild	Resolved after taking antipyretic drug
Case 3	Fever and fatigue	mild	Next day improvement
Case 4	Fever and fatigue	mild	Next day improvement
Case 5	Fever and fatigue	mild	Next day improvement
Case 6	Left axillary pain and lymphadenopathy	mild	Lymphadenopathy remain

**Table3:** Self-reported symptoms of adverse reaction to second dose of mRNA COVID-19 vaccines

N=64 (11.1%)	
Fever	58
Fatigue	21
Joint pain	12
Myalgia	11
Headache	6
Chill	6
Nausea	3
Red flash	2
Dizziness	2
Urticaria	2
Swelling	2
Cough	1
Itching	1

※multiple answers question

## Supplementary Material

PDF-Japanese
