# Supplementary material for: Adverse reactions to mRNA coronavirus disease 2019 (COVID-19) vaccine for severe acute respiratory syndrome coronavirus 2 (SARS-CoV-2) in 576 medical staff
Source: Fujita Med J. 2021 Nov 25;8(3):79–82. doi: 10.20407/fmj.2021-009 (PMC9358674; doi:10.20407/fmj.2021-009)
Supplement: Supplementary file 1 — PDF-Japanese [file fmj-8-079-s001.pdf]

当院職員576名における、新型コロナウイルス（SARS-CoV-2）に対する mRNA COVID-19ワクチンの副反応

藤田医科大学　岡崎医療センター内科学講座（呼吸器内科）准教授　林　正道[1,2]
Masamichi Hayashi M.D.,Ph.D.
藤田医科大学　岡崎医療センター内科学講座（呼吸器内科）助教 森川紗也子[1,2]
Sayako Morikawa M.D.,Ph.D.
藤田医科大学　岡崎医療センター内科学講座（呼吸器内科）助教 後藤祐介[1,2]
Yusuke Goto M.D.,Ph.D.
藤田医科大学　岡崎医療センター内科学講座（呼吸器内科）助教 吉田隆純[1,3]
Takazumi Yoshida M.D.,Ph.D.
藤田医科大学　岡崎医療センター内科学講座（呼吸器内科）助教 木村祐太郎[1,2]
Yutaro Kimura M.D.
藤田医科大学　呼吸器内科学 I 講座　教授　　今泉和良[2]
Kazuyoshi Imaizumi M.D.,Ph.D.

[1]Department of Internal Medicine, Division of Respiratory Medicine, Fujita Health University, School of Medicine, Okazaki, Aichi, Japan
[2]Department of Respiratory Medicine Ⅰ, Fujita Health University, School of Medicine, Toyoake, Aichi, Japan
[3]Department of Internal Medicine, Division of Respiratory Medicine Ⅱ, Fujita Health University, School of Medicine, Nagoya, Aichi, Japan

Corresponding author: Masamichi Hayashi, MD, PhD
Department of Respiratory Medicine Ⅰ, Fujita Health University, School of Medicine, 1-98, Dengakugakubo, Kutsukake-cho, Toyoake, Aichi 470-1192, Japan
Mail:michi@fujita-hu.ac.jp
TEL +562-93-9241

Original article

ランニングタイトル: mRNA COVID-19 ワクチンの副反応

Abstract:

Objectives:

COVID-19の流行を抑えるべく様々な対策がとられているが、効果は限定的であり、切り札として期待されているのが、ワクチンである。日本では2020年2月からワクチン接種が開始となった。しかしながら日本人における新型コロナウイルス（SARS-CoV-2）に対するmRNAワクチンの副反応報告は非常に少ない。そのため当院でワクチン接種をした職員576名における副反応を調査した。

Methods:

対象は新型コロナウイルス（SARS-CoV-2）ワクチンを接種希望した職員576名。第1回目の接種は、2021年3月8日から3月15日までの間にファイザー社の新型コロナウイルス mRNA ワクチン（商品名コミナティ筋注）にて施行した。2回目の接種は、1回目接種から21日以上間隔を開けて2021年3月29日から4月5日までに施行した。

Results:

第1回目の接種では副反応は6名に見られた。呼吸困難感1名、関節痛1名、発熱・倦怠感3名、左腋窩の痛みとリンパ節腫脹1名であった。

接種会場で対応が必要な副反応は1名であった。

第2回目の接種では副反応は64名（11.1%）に認められた。発熱58名、倦怠感21名、関節痛12名、疼痛11名、頭痛6名、悪寒6名、吐き気3名、発赤2名、めまい2名、蕁麻疹2名、腫れ2名、咳1名、かゆみ1名であった（複数回答あり）。

発熱は体温37.4度から38.9度までの範囲で認めた。

Conclusions:

COVID-19ワクチンの副反応は、軽度のものが多く、重篤なアナフィラキシーは認めなかった。副反応に注意しながら行えば、接種は十分に可能であると考えられた。

Keywords:

SARS-CoV-2、COVID-19、mRNA vaccine、Adverse reaction、Anaphylaxis

Introduction

2019年12月に新型コロナウイルス感染症（coronavirus disease 2019: COVID-19）が中国武漢で発生し、瞬く間に全世界に広がり、いくつかのピ

ークを迎えてはいるが、現在でもまだ終息する気配はない[1]。

日本でも 2020 年 2 月 3 日ダイアモンド・プリンセス号での集団発生を皮切りに短期間で広がり、現在は第 4 波となっている[2,3]。

何とか流行を抑えるべく様々な対策がとられているが、その効果は限定的である。その切り札として期待されているのが、ワクチンである。

ワクチン接種により、重症化率の低下、死亡率の低下、集団免疫の獲得による感染の終息が期待されている。

世界では 2020 年 12 月からワクチン接種が既に開始となっている。日本でも米国での第 3 相試験の結果[4]によりファイザー社（Pfizer-BioNTech）の新型コロナウイルスワクチン（商品名コミナティ）が特例承認されて、ようやく 2 月から医療従事者 4 万人に対して先行接種が開始となった。

集団免疫を得るためには、人口の 6-7 割が少なくとも 1 回はワクチンを接種する必要があるとされている。ワクチン接種を急ぐ必要があるが、2021 年 4 月 10 日の時点において、日本は人口のわずか 0.87％しかワクチン接種できておらず、米国や中国やインドや英国などの先進国と比較し残念ながら遅れていると言わざるをえない。

また副反応の報告は主に米国からのもが多く、日本からの報告はほとんどない。各国から 2 回目の接種後は副反応が増加するとの報告もある。日本でも先行接種した医療従事者においても同様の報告がなされている。

そこで、今回我々は、当院での接種希望者職員 575 名に対して 2021 年 3 月 8 日からファイザー社の新型コロナウイルスワクチンが接種開始となったため、副反応について調査をすることとした。

Methods

対象は、新型コロナウイルスワクチンを接種希望した職員 576 名。

第 1 回目の接種は、2021 年 3 月 8 日から 3 月 15 日までの間にファイザー社（Pfizer-BioNTech）の新型コロナウイルスワクチン（商品名コミナティ）にて行った。

ワクチンの入った 1 バイアルから 5 回分(5 人分)に取り分け、注射器は 1ml、針は 27G を使用した。消毒にはアルコール綿を用い、アルコールに過敏症がある人は、クロルヘキシジンを用いて皮膚消毒をした。利き腕ではないほうの肩の上腕三頭筋に 0.3ｍｌ筋肉内注射をした。

2 回目は 1 回目接種から 21 日以上間隔を開けて 2021 年 3 月 29 日から 4 月 5 日までに施行した。

予防接種不適当者は、予防接種実施規則及び新型コロナ臨時接種実施要領に規定されている、1) 新型コロナウイルス感染症に係る他の予防接種を受けたこ

とがある者、2）明らかな発熱を呈している者、3）本予防接種の接種液の成分によってアナフィラキシーを呈したことがあることが明らかな者、4）上記に該当する者のほか、予防接種を行うことが不適当な状態にある者、とした。

アナフィラキシー発生に対応するために、米国疾病予防管理局（CDC）のガイドラインに従い、重度のアレルギー反応を起こしたことがある人や、ワクチンや注射で何らかの即時型アレルギー反応を起こしたことがある人は、接種要注意者としてワクチン接種後 30 分間、それ以外の人は 15 分間、接種会場にて経過観察をした。

アナフィラキシー発生時に対応できる体制を確保するために、アドレナリン製剤など、救急処置に必要な物品を備えた緊急カートや搬送用のストレッチャーやベッドを準備した。

ワクチンの準備は薬剤師が行った。医師 2 名が予診票を確認し、看護師 2 名がワクチン接種、看護師 1 名が経過観察を行った。予診票の事前確認や受付などは事務員 4 名で行った。接種会場に間隔を空けて椅子を 30 名分用意し、椅子に座った状態で経過観察を行った。

経過観察後の副反応は自己申告制で各部署の上司に報告し、感染対策室（ICT）が症状について詳細な問診をした。副反応と考えられた事例に関しては「副反応疑い報告」として厚生労働省に報告をした。

Results

接種希望者の年齢は平均34.9歳（21～69歳、中央値32歳）、性別は女性396人、男性180人であった（Table 1）。既往歴は、高血圧症15 名、高脂血症7名、子宮内膜症4名、糖尿病4名、月経困難症3名、甲状腺機能亢進症3名、卵巣嚢腫2名、うつ病2名、緑内障2名、てんかん1名、全身性エリテマトーデス（SLE）1名、前立腺肥大症1名、関節リウマチ1名、ジストニア1名、閉塞性睡眠時無呼吸症候群（OSA）1名、片頭痛1名、クローン病1名、胃食道逆流症1名、胆石症1名であった（Table 1）。アレルギーの既往は合計36名で、花粉症が7名、気管支喘息が6名、アトピー性皮膚炎が5名、アレルギー性鼻炎が2名、甲殻類が3名、卵・乳製品が2名、他にぶり、桃、キウイ、シイタケ、そば、桃が各1名ずつ、抗生剤等内服薬に関するものが5名であった。アナフィラキシーなど重いアレルギー症状の既往はなかったが、インフルエンザワクチンで発熱の副反応が3名、子宮頸がんワクチンで発熱の副反応が2名であった（Table 1）。

第 1 回目の接種では副反応は 6 名（1.0%）に見られた。呼吸困難感 1 名、関節痛 1 名、発熱・倦怠感 3 名、左腋窩の痛みとリンパ節腫脹 1 名であった。

呼吸困難感を訴えた職員は、48 歳女性で、気管支喘息の既往歴があり、接種 10 分後に呼吸困難感、倦怠感、頭痛を訴え、血圧 133/67mmHg、脈拍数 65 回

/分、SpO_2 99%であり、口角に膨疹を軽度認めた。ブライトン分類[5]におけるアナフィラキシーに該当しなかったが、補液と抗ヒスタミン薬の投与によって速やかに症状は改善した。

第 2 回目の接種では副反応は 64 名（11.1%）に認められた。発熱 58 名、倦怠感 21 名、関節痛 12 名、疼痛 11 名、頭痛 6 名、悪寒 6 名、吐き気 3 名、発赤 2 名、めまい 2 名、蕁麻疹 2 名、腫れ 2 名、咳 1 名、かゆみ 1 名であった（複数回答あり）。

接種会場にて処置が必要な副反応は、呼吸困難に対して点滴処置を行った 1 名のみであった。この職員のみ安全を期して 2 回目の接種は行わなかった。

2 回目の接種後の副反応は 64 名（11.1%）に認められ、1 回目の副反応 6 名（1.0%）から 10.6 倍に増加した。

発熱は当日の夕方から翌日に体温 37.4 度から 38.9 度までの範囲で認められた。

Discussion

当院職員においては、2 回目接種後に副反応、特に発熱が増加した。現在はコロナ渦であり、発熱があると感染症疑いとなり職員は出勤できなくなる。多数の職員が出勤できなくなると必然的に病院としての機能不全に陥る可能性がある。これを防ぐために当院では、予測されていた 2 回目接種による発熱や関節痛に対して、予め職員には解熱鎮痛剤を使用し、翌日解熱し症状が改善していれば出勤可能とした。今後、他の医療機関でも同様に対策が必要と考えられた。

米国のCDCはv-safeという予防接種副反応報告システムを用いてワクチン接種後初めの7日間、6週目まで毎週、それから3・6・12か月後に副反応の調査を21,843,033名に対して行っている[6]。2021年1月14日時点で、全体では疼痛が70.7%、倦怠感が33.4%、頭痛が29.4%、筋肉痛が22.8%、悪寒が11.5%、発熱が11.4%、腫れが11.0%、関節痛が10.4%、吐き気が8.9%に認められたと報告されている[6]。ファイザー社(Pfizer-BioNTech)のワクチン1回目の副反応は疼痛が67.7%、倦怠感が28.6%、頭痛が25.6%、筋肉痛が17.2%、悪寒が7.0%、発熱が7.4%、腫れが6.8%、関節痛が7.1%、吐き気が.0%であり、2 回目の副反応は疼痛が74.8%、倦怠感が50.0%、頭痛が41.9%、筋肉痛が41.6%、悪寒が26.7%、発熱が25.2%、腫れが26.7%、関節痛が21.2%、吐き気が13.9%と報告されており[6]、2 回目の副反応が増加している。

米国における CDC と FDA が共同で行っている予防接種副反応報告システム VAERS（Vaccine Adverse Event Reporting system ）ではファイザー社（Pfizer-BioNTech）のワクチンが接種された 7,307 名に対して副反応の報告

が行われた。2021 年 1 月 18 日時点の全体での副反応は、頭痛 21.2%、倦怠感 16.3%、めまい 15.2%、吐き気 13.9%、悪寒 13.5%、発熱 13.2%、疼痛 13.1%、注射部の痛み 9.8%、四肢の痛み 8.4%、呼吸困難感 7.3%が副反応として報告されている。

当院における副反応は自己申告制のためか、注射部位の疼痛や腫れ、倦怠感など予測されていたものは、少なかった。最も多く認められた発熱に関しては、諸外国の報告と同様に認められた。またアナフィラキシーは認めなかった。

米国において2020年12月14日から12月23日までの期間に190万回接種し、21件のアナフィラキシーが報告されている。これは100万回接種に換算すると11.1件の報告となる[7]。

同じく米国において2020年12月14日から2021年1月18日までの期間に994万回接種し、47件のアナフィラキシーが報告されている。これは100万回接種に換算すると4.7件の報告となる。74%が接種後15分以内、90%が30分以内に発生しており、80%はアレルギーの既往のある人であった[8,9]。

英国において2020年12月9日から2021年2月28日までの期間に1150万回接種し、214件のアナフィラキシーが報告されている。これは100万回接種に換算すると18.6件の報告となる[10]。

米国の医療ネットワークの職員64,900人を対象にした研究では、ファイザー社製ワクチン接種25,929人において、自己報告で506件（1.95%）にアレルギー反応、7件（1万人あたり2.7件）にアナフィラキシーが認められた[11]。

ワクチンによるアナフィラキシー頻度は、一般的に100万回接種あたり1.3件とされており、これまでより多いことが懸念されている[12]。

日本では、副反応を確認した医療機関が、予防接種法及び医薬品医療機器等法に基づき、医薬品医療機器総合機構（PMDA）に対して副反応疑い報告を速やかに実施し、厚生労働省と情報共有することになっている。

日本では、2021年2月17日から2021年3月11日までに181,184回接種し、37件のアナフィラキシーが報告されている。これは10万人当たり20.4人に相当し、100万回接種に換算すると204件の報告となる[13,14]。

国内の報告件数は、欧米と比較して非常に多いと考えられるが、現時点での日本の報告数は医療機関からの報告数そのものであることから、情報を精査し、ブライトン分類ではアナフィラキシーに該当しない可能性がある。

日本アレルギー学会からの指針では、COVID-19ワクチンにアジュバントや保存剤は添加されていないが、ファイザー社とモデルナ社のmRNAワクチンは有効成分であるmRNAが封入されている脂質ナノ分子を形成する脂質二重膜の水溶性を保持するためにポリエチレングリコール（polyethylene glycol: PEG）が使用されており、これがアナフィラキシーの原因と考えられてい

る。ただし、現時点ではワクチンの主成分である二本鎖RNAに対する特異的IgE産生の可能性が否定されている訳ではない[15]とされている。

今後、日本での副反応報告が蓄積されてくると考えるが、これを待たずに既に全国でワクチン接種が始まっている。

mRNAワクチンは従来のワクチンとは異なる技術を用いて開発・製造されており、副反応については不明な点も多い。

重度の過敏症であるアナフィラキシーをきたす頻度が従来のワクチンよりも高いことが報告されているが、アナフィラキシーの症状と対処自体は他の原因によるものと変わらず、新型コロナウイルスワクチンによるアナフィラキシーも適切な対処により回復する。

これから医療従事者に対する接種がさらに広がる予定だが、4 月 12 日から一部の高齢者に対してワクチン接種が開始されている。

日本における新型コロナワクチンの副反応の報告はほとんどないため、当院における副反応報告は、今後のワクチン接種に際して、非常に重要な情報になると考える。

現場で対応をしている医療従事者が混乱せず、適切な処置が行えるために、今回の報告が少しでも役に立てばと考える。

Conclusion

COVID-19 ワクチンは、副反応は 2 回目に多くみられたが、軽度のものが多く、重篤なアナフィラキシーは認めなかった。

欧米からの報告にあるように副反応に注意しながら行えば、接種は十分に可能であると考えられた。

今後も最新の副反応情報を確認し、ワクチン接種を適切に行う必要があると考えられた。

Study limitations

今回の副反応の報告結果は単施設での検討であり、また自己申告制である。詳細な副反応情報を得るためには、米国のように電話やウェブなどを利用し調査するようなシステムの構築が必要と考えられた。

また今後は症例数を増やし副反応の報告を蓄積するとともに、他施設との共同研究が必要であると考えられた。

Conflict of Interest

This study was performed without financial support and there are no conflicts of interest to declare.

Masamichi Hayashi has received honoraria for lectures from KYORIN Pharmaceutical Co. Ltd. and GlaxoSmithKline K.K..

Funding Sources

None.

Acknowledgments

We would like to thank the doctors from the Department of Internal Medicine (Respiratory Medicine II), Fujita Health University.

References

1. Guan W, et al. Clinical characteristics of 2019 novel coronavirus infection in China. N Engl J Med 2020;382:1708-20
2. Matsunaga N, Hayakawa K, Terada M, et al. Clinical epidemiology of hospitalized patients with COVID-19 in Japan: Report of the COVID-19 REGISTRY JAPAN. Clin Infect Dis 2020:ciaa1470.
3. Aki S Sakurai A, Sasaki T, Kato S, Hayashi M, Tsuzuki SI, Ishihara T, Iwata M, Morise Z, Doi Y. Natural History of Asymptomatic SARS-CoV-2 Infection. N Engl J Med 2020; 383:885-6
4. Polack FP, Thomas SJ, Kitchin N, et al. Safety and efficacy of the BNT162b2 mRNA Covid-19 vaccine. N Engl J Med. 2020;383(27):2603-2615.
5. Public Health England. Guidance COVID-19: the green book, chapter 14a Coronavirus (COVID-19) vaccination information for public health professionals. <https://www.gov.uk/government/publications/covid-19-the-green-book-chapter-14a > (Accessed April,10,2021)
6. Shimabukuro TT. COVID-19 vaccine safety update-CDC. January 27,2020 <https://www.cdc.gov/vaccines/.../06-COVID-Shimabukuro.pdf (Accessed April,10,2021)
7. CDC COVID-19 Response Team; Food and Drug Administration. Allergic Reactions Including Anaphylaxis After Receipt of the First Dose of Moderna COVID-19 Vaccine - United States, December 21, 2020-January 10, 2021. MMWR Morb Mortal Wkly Rep. 2021;70:125-9.<https://www.cdc.gov/mmwr/volumes/70/wr/mm7002e1.htm?s_cid=mm7002e1_w> (Accessed April,10,2021)

8. Centers for Disease Control and Prevention. COVID-19 vaccine safety update;2021.<https://www.cdc.gov/vaccines/acip/meetings/downloads/slides-2021-02/28-03-01/05-covid-Shimabukuro.pdf> (Accessed April,10,2021)
9. Shimabukuro T, et al. Reports of Anaphylaxis After Receipt of mRNA COVID-19 Vaccines in the US JAMA 2021;325:1101-2.
10. MHRA Coronavirus Vaccine - summary of Yellow Card reporting <https://assets.publishing.service.gov.uk/government/uploads/system/uploads/attachment_data/file/966633/Coronavirus_vaccine-summary_of_Yellow_Card_reporting.pdf> (Accessed April,10,2021)
11. Blumenthal KG, Robinson LB, Camargo CA Jr, Shenoy ES, Banerji A,Landman AB, Wickner P. Acute Allergic Reactions to mRNA COVID-19Vaccines. JAMA 2021;20;325:1562-5.
12. McNeil MM, Weintraub ES, Duffy J, et al. Risk of anaphylaxis after vaccination in children and adults. J Allergy Clin Immunol. 2016;137(3):868-78.
13. Ministry of Health, Labour and Welfare. Shingata korona wakuchin no fukuhanno utagai hokoku nitsuite. (in Japanese). <https://www.mhlw.go.jp/stf/seisakunitsuite/bunya/vaccine_sesshujisseki.html> (Accessed April,10,2021)
14. MHRA Coronavirus Vaccine - summary of Yellow Card reporting <https://www.mhlw.go.jp/stf/seisakunitsuite/bunya/vaccine_hukuhannou-utagai-houkoku.html> (Accessed April,10,2021)
15. Japanese Society of Allergology. Shingata korona uirusu wakuchin sesshu ni tomonaujudo no kabinsho(anafuirakishi nado)no kanri・shindan・chiryo. (in Japanese). <https://www.jsaweb.jp/modules/about/index.php?content_id=81 > (Accessed April,10,2021).
